# The future and the female academic leader: advancing student engagement

**DOI:** 10.3389/fpsyg.2014.00377

**Published:** 2014-04-29

**Authors:** Carl Senior, Christopher Howard, Rowena Senior

**Affiliations:** ^1^School of Life and Health Sciences, Aston UniversityAston Triangle, Birmingham, UK; ^2^School of Psychology, Derby UniversityDerby, UK; ^3^Centre for Learning, Innovation and Professional Practice, Aston UniversityBirmingham, UK

**Keywords:** academic leadership, email, technology, student engagement, student retention

The ever-changing face of academic leadership demands constant consideration and as such there is clearly much interest within the community at large to examine this most important of academic roles. To be truly effective the academic leader must be a unique animal who goes beyond merely ensuring that departmental budgets are aligned at the end of the fiscal year but use an ever evolving technological environment to lead in all aspects of teaching, research as well as administrative duties in support of their respective department. In this environment leaders not only need to be aware of, but demonstrate proficiency, in the use of MOOCs, cloud based computing and even efficient use of email software (Huang, 2001; Ruby, 2013). Such a task may seem to be quite unnerving to the new entrants to the field—this is especially relevant for the female academic who may not readily adopt such emerging technologies (Venkatesh and Morris, 2000). However, in a sector where almost all new entrants expect to achieve a leadership position at some point it is important to highlight the current orthodoxy with regards to achieving such a position and any possible mediating role that technology may play. Current research endeavors that highlight the moderating effects of gender in achieving leadership positions within academia are discussed here. It is argued that such research takes a limited perspective on both the roles required with academia but also the unique and very important contributions that female leaders can provide to students. Here, it is clear that the unique collegial and social manner of a female style of leadership that is often seen with managers in industry is an ideal trait for leadership within academia especially with regards to the most important aspect of academia, and often the most overlooked, and that is the student (Eagly and Karau, 1991). Student engagement is a key determinant for improving student motivation, how students approach learning and academic success (Lazaros and Davidson, 2013). It is argued here that technology, particularly the use of email, can play an important role in providing students with access to the unique skill set of certain academic leaders.

Probably the largest examination of the effects of gender on academic leadership is seen with the recent work by Parker and Welch (2013). Here, an extensive analysis of a large scale dataset was carried out to examine how an individual's professional network, an individual's scientific ability or the gender of an individual independently predicted academic leadership at the level of the research center, the level of the university administrator or even at the position of overarching leadership at the level of the specific scientific discipline (Parker and Welch, 2013). This study is indeed a comprehensive analysis and, while not without its limitations, revealed that scientific productivity and reputation predicted leadership roles at the center level but this was moderated by gender. The study also showed a paucity of female academics at the research center and administrative leadership level but found that females held significantly more leadership positions at the wider discipline level. These are the positions that are generally high profile and serve to act as inspirational role models for students who wish to enter that particular field. However, even though Parker and Welch (2013) was clearly an important study they did not fully address what could be considered the most important aspect of academic leadership and that is understanding how interactions between the leaders of academic institutions and the student body can improve the entire learning environment (Smith and Hughey, 2006). Here, it is argued that use of technology can help to ensure that the students fully interact with female leaders within academia and benefit from their style of leadership.

There is no doubt that engaging with students at a social level enhances their overall experience during their course of study and even predicts their actual performance on assessments (Tinto, 1975; Kuh, 1993; Koljatic and Kuh, 2001; Smith et al., 2005). Indeed, we have previously found that undergraduate students consider the quality of their engagement with staff to be the single most important factor in driving their engagement with their programme of study (Towl and Senior, 2010). However, while it is abundantly clear that students do want to have more and more immediacy with their teachers it is not clear if they consider teachers who are discipline leaders as more effective in meeting this requirement.

A female leadership style is typically exemplified by a collegial, friendly and democratic style of social interaction (Bartol, 1974; Eagly and Carli, 2003)^1^. This is the very style of social leadership that students expect. Yet there is seems to be a misalignment occurring between the positioning of leaders to roles where they are likely to be the most effective. As noted above female academics are less likely to find themselves in positions of leadership at the research center or administrative level. However, these are the very roles that are likely to involve interaction with students, which is crucial as students who feel they have a say in their learning or have opportunity to participate in debate, which reflects the democratic approach often employed by female academic leaders, drives higher levels of student engagement (Exeter et al., 2010). This is problematic as the current orthodoxy in academia misaligns the effective leadership abilities of female style of leadership. However, it is with the judicious use of technology to interact with students that academic leaders can indeed play a pivotal role with enhancing the student learning experience.

Email, as a means of communication, is now ubiquitous and it is fair to say that all students enrolling on a programme of higher education are provided with an email account and access to computing facilities by which to use it (Huang, 2001). However, and perhaps more importantly, email exchanges between student and teacher can actually serve a more social role that facilitates the immediacy of staff (Bloch, 2002). Such email driven immediacy is considered in a more positive light by the student cohort and also predicts improvement in subsequent assessments (Sheer and Fung, 2007). While the prevalence of email exchange between student and staff member is considered in a positive light by the student cohort such perceptions are always in the service of the “nurturing, open, nonthreatening, and respectful” relationships with staff members (Anderson and Carta-Falsa, 2002, p. 134). Students simply need to feel that they are respected members of the learning community before they start to develop an independent approach to their learning.

Such community affiliation can be readily developed by the friendly and collegial approach that is diagnostic of a female style leadership. Indeed, these relationship-based behaviors are often considered to be at the very core of effective leadership (Lowe et al., 1996; Bommer et al., 2004). As female academics tend to be discipline level leaders, a position that is traditionally removed from much student facing contact, it would seem that email interaction may be an effective means to ensure that students benefit from the unique collegiality of certain discipline leaders and develop stronger ties to the immediate learning community. The students will get ready access to those leaders who may play an inspirational role model in helping them engage with their studies and shaping their long-term aspirations. The development of such email assisted immediacy should in turn start to see a shift away from the current model of management that has evolved in academia where large groups of students end up having little contact with the discipline lead (Hubel, 2009). In today's academic environment, with the ever-growing list of demands placed on its leaders, it is intriguing to suggest that that by merely using e-mail we may see a return to the model of practice within academia where experience is shared universally and not communicated in a predominantly hierarchical fashion.

## Conflict of interest statement

The authors declare that the research was conducted in the absence of any commercial or financial relationships that could be construed as a potential conflict of interest.

## Footnotes

^1^It is worth noting that such a style of leadership is not gender specific and can be demonstrated by both male and female leaders (See Fitzgerald, 2014 for an excellent discussion on this area).
